# Measurement of Glomerular Filtration Rate Using Multiphasic Computed Tomography in Patients With Unilateral Renal Tumors: A Feasibility Study

**DOI:** 10.3389/fphys.2019.01209

**Published:** 2019-09-19

**Authors:** Tingting Wang, Yi Xu, Wangyan Liu, Pengfei Shao, Qiang Lv, Guanyu Yang, Lijun Tang

**Affiliations:** ^1^Department of Nuclear Medicine, The First Affiliated Hospital, Nanjing Medical University, Nanjing, China; ^2^Department of Heart Center, The First Affiliated Hospital, Auhui Medical University, Hefei, China; ^3^Department of Radiology, The First Affiliated Hospital, Nanjing Medical University, Nanjing, China; ^4^Department of Urology, The First Affiliated Hospital, Nanjing Medical University, Nanjing, China; ^5^Laboratory of Image Science and Technology, School of Computer Science and Engineering, Southeast University, Nanjing, China

**Keywords:** tomography, kidney neoplasms, volume measurements, renal function, glomerular filtration rate

## Abstract

**Objectives:**

This study was to assess the feasibility of a modified multiphasic CT scan protocol combined with homemade software measurements of glomerular filtration rate (CT-GFR) and explore the effect of renal tumor volume on the calculation of CT-GFR.

**Materials and Methods:**

Prospective observational study comparing three methods of GFR measurement from February 2017 to December 2017, 91 patients with unilateral renal tumor underwent both a modified multiphasic CT scans of kidney and serum creatinine (Scr) tests preoperatively, of which 15 cases underwent additional radionuclide examination. Total and split CT-GFR, with or without renal tumor, were quantified by the homemade software in early and late renal parenchymal phases, respectively. The volume of renal tumor was quantified by the homemade software. Correlation and difference between CT-GFR and traditional methods of GFR measurement, including estimated GFR (eGFR) from Scr concentration and split GFR using of radionuclide examination (R-GFR), were performed.

**Results:**

There is a strong correlation between CT-GFR with renal tumor and eGFR (*r* = 0.90, *p* < 0.001) in early renal parenchymal phase. The relative CT-GFR in early renal parenchymal phase was highly correlated with the relative R-GFR (*r* = 0.88, *p* < 0.001). Renal tumor volume significantly correlated with the value of CT-GFR that determined by subtracting the CT-GFR measurement without renal tumor from CT-GFR measurement with renal tumor (*r* = 0.89, *p* < 0.001).

**Conclusion:**

A modified multiphasic CT scan protocol combined with homemade software might be an alternative technique for the evaluation of renal function for the patients with unilateral renal tumor.

## Introduction

Glomerular filtration rate (GFR) is still considered as the best indicator in clinical assessment of renal function. Inulin clearance is considered a gold standard for total GFR determination. But it can not be applied widely in clinical practice because of its technical complexity and time-consuming procedure (Ingelfinger and Marsden, 2013). Split GFR is most commonly measured by radionuclide examination (R-GFR). However, radionuclide examination has several disadvantages including exposure to radioisotopes, short length of patient isolation following the study, and can not estimate the effect of volume of renal tumor on the accuracy of renal function measurement (Kerl and Cook, 2005). In light of the limitations of traditional methods of GFR measurement, assessment of both morphologic and functional information about the kidneys using CT has been reported (Yokoyama et al., 1983; El-Ghar et al., 2004; Kwon et al., 2015; Yuan et al., 2017). But most of the studies mainly focused on patients with renal artery stenosis, ureteral obstruction, or renal atrophy. Few studies have reported GFR measurement using CT in patients with renal tumor, and few studies have explored the effect of the tumor itself on renal function.

The incidence and detection of renal tumor rapidly increasing with the widespread use of various imaging modalities and with the aging of population (Chow et al., 1999). GFR measurement of the patients with renal tumor pre- and post-nephron-sparing surgery such as laparoscopic partial nephrectomy may be important in treatment strategy decision and prognosis evaluation. However, the ability to obtain GFR using CT data for patients with renal tumor and the effect of the volume of tumor itself on renal function are still unknown. In the present study, we introduced a modified multiphasic CT scan protocol of whole kidney in combination with a homemade software for measuring GFR based on the technique of Patlak plot. The purpose of this study was to investigate the feasibility and accuracy of GFR derived from CT images (CT-GFR) of patients with unilateral renal tumor.

## Materials and Methods

### Study Population

This prospective study was approved by our local ethic institutional review board, and written informed consent was obtained from all patients before study entry. From February 2017 to December 2017, 95 patients with unilateral renal tumor were enrolled in our study. Four subjects were excluded due to inability to follow breath-hold commands. All patients had no acute renal disorders. The remaining 91 patients with 182 kidneys were enrolled in data analysis, including 58 males and 33 females (mean age 58 ± 12 years, range 28–81 years). All the patients underwent both multiphasic CT scans of kidney and serum creatinine (Scr) tests before surgery. The Hematocrit (Hct) and Scr value of all patients were determined by blood sample obtained prior to CT examination within 2 days. Fifteen patients underwent radionuclide imaging with ^99m^Tc-diethylenetriaminepentacetic acid (^99m^Tc-DTPA) before surgery.

### eGFR Measurements

Estimated GFR was estimated from Scr concentration by using the 2009 EPI-CKD creatinine equation (Sabanayagam et al., 2009). Scr concentrations were measured by IDMS-traceable calibrator (Kehua Bio-Engineering Co., Ltd. Diagnostics). The mean of Scr in all the study population was 0.77 ± 0.17 mg/dl, range 0.36–1.37 mg/dl.

### Relative Split R-GFR Measurements

^99m^Tc-diethylenetriaminepentacetic acid was given in the antecubital vein. The total injected dose was determined by subtracting the post-count from the pre-count. The individual R-GFR was automatically calculated using the Gates method according to patient’s weight, height, and the kidney contour (Siemens E.CAM, Siemens) (Gates, 1982, 1983). The relative split R-GFR of a kidney, expressed as a percentage of total renal function, was calculated by dividing unilateral R-GFR with total R-GFR.

### Multiphasic CT Scan Protocol and CT-GFR Measurements

#### Multiphasic CT Scan Protocol

All CT examinations were performed on a first-generation dual-source CT scanner (Somatom Definition, Siemens). The scan was performed with the following parameters: tube voltage 120 kV, effective tube current 200–250 mAs, collimation width 64 × 0.6 mm, effective slice thickness 0.75 mm. The scan range covered the whole kidneys.

**FIGURE 1:**
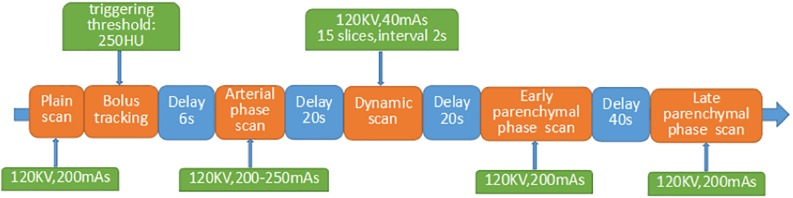
Flowchart of a modified multiphasic CT scan protocol.

The multiphasic CT scan protocol (Figure 1) consisted of a plain scan followed by three contrast medium-enhanced examinations which were an arterial phase, an early parenchymal phase, and a late parenchymal phase, respectively. In addition, between arterial phase and early parenchymal phase, one dynamic scan (15 scans, scan interval 2 s, tube potential 120 kVp, tube current 40 mAs, slice thickness 10 mm) was initiated at the level of renal hilum. After the plain scan, the arterial scan was triggered by the bolus tracking technique after 100 ml of contrast injection (Ultravist 370, Schering) in the antecubital vein at a velocity of 5 ml/s. The region of interest (ROI) was placed within the descending aorta at the level of renal hilum and the triggering threshold was set as 250 HU.

#### CT-GFR Measurements

The GFR of kidney measured by CT is based on the method of two point of Patlak plot (Patlak et al., 1983; Tsushima, 1999). The Patlak plot method is described by a two-compartment model with unilateral tracer flow from compartment 1 into compartment 2. In our study, compartment 1 models the vascular space and compartment 2 models the nephron space. Image data acquired from the multiphasic CT scan were transferred to a homemade software for further processing.

At first, contours of both kidney’s parenchyma in arterial phase including cortex and medulla were segmented automatically by our software (Supplementary Figure 1). The structures in the renal hilum such as renal pelvis, vessels, and fatty tissue were excluded manually.

**FIGURE 2:**
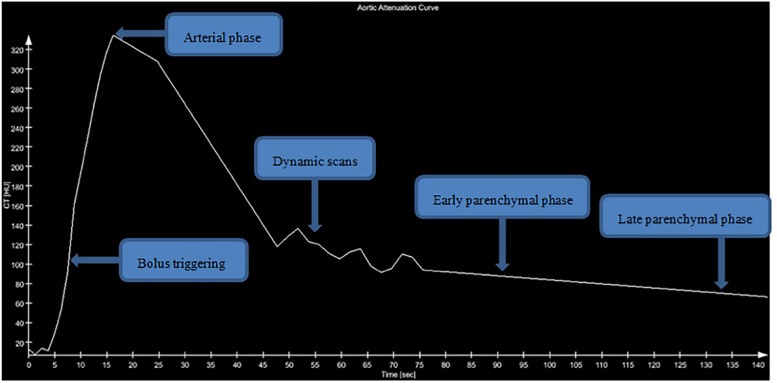
Aortic attenuation curve. Graph shows aortic attenuation curve of a 35-year-old man with unilateral renal tumor. Five clusters of data points were measured with CT. The area under the curve was determined graphically.

Secondly, aortic time attenuation curve (TAC) was determined by the CT attenuation values of circular ROI in abdomen aorta at the level of the renal hilum. In our study, the aortic TAC was drawn from multiple time points including plain scan phase, bolus triggering, arterial phase, dynamic scans, early parenchymal phase, and late parenchymal phase (Supplementary Figure 4). Figure 2 showed the aortic TAC by the homemade software.

Thirdly, registration of images including plain scan, arterial, and early or late parenchymal phases were performed. Both the net attenuation value of parenchymal and the volume of each kidney were automatic calculated by the homemade software. The individual CT-GFR adjusted for body surface area (BSA) of split kidney, with tumors or without tumors, was automatically calculated by the homemade software after entering the patient’s data of weight, height, and Hct values. Additionally, total CT-GFR was the sum of bilateral kidney CT-GFR values. The CT-GFR of every patient was independently measured using the homemade software by two observers, respectively.

### Statistical Analysis

Statistical analysis was performed using SPSS 23.0 and MedCalc 13.0. Quantitative data were tested for homogeneity of variance by the Kolmogorov–Smirnov one-sample test. Continuous variables were expressed as means ± standard deviation (SD). Paired *t-*test and linear correlation analysis were performed. The agreement was evaluated using Bland–Altman analysis. *p-*values < 0.05 were considered statistically significant.

## Results

### GFR Measurements by Three Methods

**FIGURE 3:**
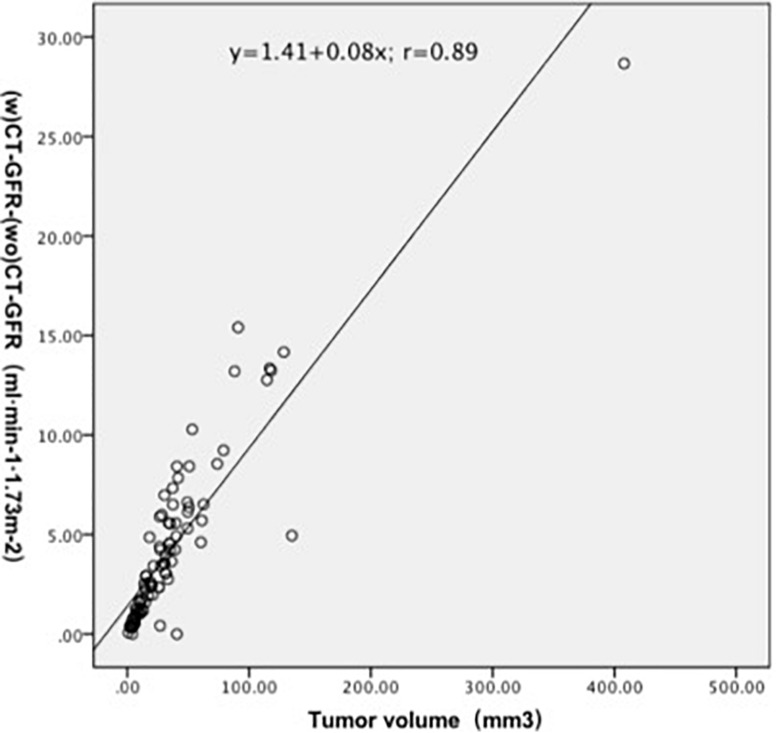
Volume of renal tumor significantly correlated with the value of CT-GFR that determined by subtracting the CT-GFR measurement without renal tumor from CT-GFR measurement with renal tumor (*r* = 0.89, *p* < 0.001).

All patients had unilateral renal tumor, 42 at left side and 49 at right side. The tumor volume in patients ranged from 0.78 to 407.94 cm^3^, with a median of 26.35 cm^3^. Study subject characteristics are summarized in Supplementary Table 3. Quantitative data of all GFR turned out to have a normal distribution according to results of the Kolmogorov–Smirnov one-sample test. The Hct among all the study population was 0.40 ± 0.04, ranged from 0.29 to 0.50. In the present study, the additional radiation exposure caused by 15 dynamic scans with 40 mAs is estimated to be 0.51 mSv, which accounts for about 3% of the radiation dose of the protocol. Table 1 lists the values of GFR measurement by CT, CKD-EPI, and radionuclide examination. Figure 3 illustrates the correlation between renal tumor volume and the value of CT-GFR that determined by subtracting the CT-GFR measurement without renal tumor from CT-GFR measurement with renal tumor (*r* = 0.89, *p* < 0.001).

**TABLE 1:** Results of statistical of GFR by three methods $\left(\bar{x}+s\right)$.

		**Tumor-bearing kidney**		**Non-tumor-bearing kidney**		**Total**
				
**GFR measurement**	**Phase of renal parenchymal**	**Absolute value (ml**.**min^–1^**.**1.73 m^–2^)**	**Relative value (%)**	**Absolute value (ml**.**min^–1^**.**1.73 m^–2^)**	**Relative value (%)**	**Absolute value (ml**.**min^–1^**.**1.73m^–2^)**
CT-GFR	Early	47.18 ± 9.54	51.20 ± 2.88	44.47 ± 8.37	48.80 ± 2.88	91.65 ± 17.02
(w)	Late	38.10 ± 8.71	51.36 ± 3.03	35.66 ± 7.62	48.64 ± 3.03	73.76 ± 15.61
CT-GFR	Early	42.94 ± 9.02	50.53 ± 3.08	44.47 ± 8.37	50.96 ± 2.98	87.41 ± 16.59
(wo)	Late	34.66 ± 8.03	49.21 ± 2.97	35.66 ± 7.62	50.79 ± 2.97	70.32 ± 15.05
R-GFR		52.03 ± 11.27	50.03 ± 2.75	52.99 ± 14.23	49.97 ± 2.75	103.68 ± 24.01
eGFR						95.18 ± 15.94

*CT-GFR(w), quantitative glomerular filtration rate with tumor based on the method of two point of Patlak plot; CT-GFR(wo), quantitative glomerular filtration rate without tumor based on the method of two point of Patlak plot; R-GFR, quantitative glomerular filtration rate based on the method of radionuclide examination; eGFR, quantitative glomerular filtration rate based on the equation of EPI-CKD.*

### Comparison Between Total CT-GFR and eGFR

**FIGURE 4:**
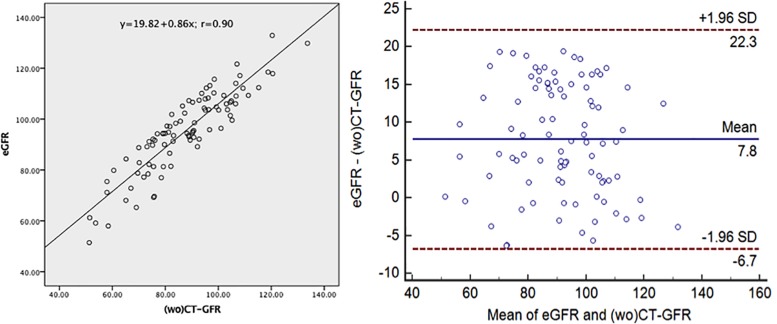
Correlation and concordance between the total CT-GFR without tumor in early renal parenchymal phase and eGFR. (wo)CT-GFR, quantitative glomerular filtration rate (without tumor) in early renal parenchymal phase based on the method of two point of Patlak plot; eGFR, quantitative glomerular filtration rate based on the equation of EPI-CKD.

Table 2 lists the correlation and difference between total CT-GFR and eGFR. There was a high correlation between the total CT-GFR without tumor in early renal parenchymal phase and eGFR (*r* = 0.90, *p* < 0.001). The difference between eGFR and total CT-GFR without tumor in early renal parenchymal phase was 7.98 ± 7.43 ml.min^–1^.1.73 m^–2^ (*p* < 0.001). Figure 4 illustrates the correlation and concordance from the correlation analysis and Bland–Altman test between the total CT-GFR without tumor in early renal parenchymal phase and eGFR. A limits of agreement plot showed that a mean difference of 7.8 ml.min^–1^.1.73 m^–2^ with 95% CI −6.7 to 22.3 ml.min^–1^.1.73 m^–2^. Values of split GFR of tumor-bearing kidney were significantly different between with and without renal tumor (Supplementary Table 1).

**TABLE 2:** Results of statistical comparison between CT-GFR and eGFR.

		**Paired *t-*test difference**			**Correlation analysis**	
			
**GFR measurement**	**Phase of renal parenchymal**	***x*^∗^ (*n* = 70) (ml**.**min^–1^**.**1.73 m^–2^)**	***t-*value**	***p-*value**	***r-*value**	***p-*value**
CT-GFR	Early	7.77 ± 7.40	10.01^δ^	<0.001^δ^	0.90^δ^	<0.001^δ^
(wo)	Late	24.86 ± 9.55	24.83^δδ^	<0.001^δδ^	0.81^δδ^	<0.001^δδ^
eGFR		…	…	…	…	…
CT-GFR	Early	3.53 ± 8.25	4.08^θ^	<0.001^θ^	0.88^θ^	<0.001^θ^
(w)	Late	21.42 ± 10.13	20.16^θθ^	<0.001^θθ^	0.79^θθ^	<0.001^θθ^

*^∗^Data are $\bar{x}$ ± standard deviation. ^δ^Data represent results of the comparison between eGFR and CT-GFR(wo) in early renal parenchymal phase. ^δδ^Data represent results of the comparison between eGFR and CT-GFR(wo) in late renal parenchymal phase. ^θ^Data represent results of the comparison between eGFR and CT-GFR(w) in early renal parenchymal phase. ^θθ^Data represent results of the comparison between eGFR and CT-GFR(w) in late renal parenchymal phase. CT-GFR(w), quantitative glomerular filtration rate with tumor based on the method of two point of Patlak plot; CT-GFR(wo), quantitative glomerular filtration rate without tumor based on the method of two point of Patlak plot; eGFR, quantitative glomerular filtration rate based on the equation of EPI-CKD.*

### Comparison Between Relative Split CT-GFR and Relative R-GFR of Kidneys

**FIGURE 5:**
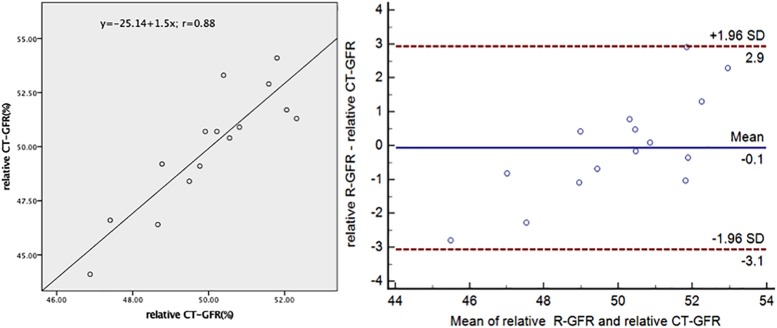
Correlation and concordance between the relative R-GFR and relative CT-GFR of split kidney of non-tumor-bearing in early renal parenchymal phase. CT-GFR, quantitative glomerular filtration rate based on the method of two point of Patlak plot; R-GFR, quantitative glomerular filtration rate based on the radionuclide examination.

Correlation and difference between relative CT-GFR and relative R-GFR of non-tumor-bearing and tumor-bearing kidneys of 15 patients were listed in Supplementary Table 2. The relative GFR of split kidney, expressed as a percentage, calculated by split CT-GFR value divided by total CT-GFR and the same as in calculating relative R-GFR. There was a strong correlation between relative CT-GFR and relative R-GFR in early renal parenchymal phase (*r* = 0.88, *p* < 0.001). There was no significant difference between relative CT-GFR and relative R-GFR of split kidney in early parenchymal phase (*r* = 0.88, *p* > 0.05). Figure 5 illustrates the correlation and concordance from the correlation analysis and Bland–Altman test between relative CT-GFR and relative R-GFR of non-tumor-bearing kidney. A limits of agreement plot showed that a mean difference of −0.1 percentage points with 95% CI −3.1–2.9.

### Agreement of CT-GFR Measurement

Bland–Altman test shows the results of CT-GFR measurement by the homemade software from the of two experienced radiologists. High levels of agreement of this software has been proved using inter-observer comparison (Supplementary Figure 2).

## Discussion

A preoperative evaluation of GFR of patient with tumor is very important in clinical decision-making and treatment response assessment, especially value of split GFR. Within this study, we introduced a practical approach based on CT for the estimate of split and total renal function preoperatively. The greatest advantage of our approach is that it can calculate split and total GFR with and without renal tumor. The effect of with or without renal tumor on the measurement of CT-GFR values was investigated in our study. Renal tumor volume significantly correlated with the value of CT-GFR that determined by subtracting the CT-GFR measurement without renal tumor from CT-GFR measurement with renal tumor (*r* = 0.89, *p* < 0.001). There was a statistically significant difference between the values of CT-GFR with renal tumor and the values of CT-GFR without renal tumor. Values of CT-GFR measured without renal tumor showed a better correlation with eGFR (*r* = 0.90, *p* < 0.001) than values of CT-GFR measured with renal tumor (*r* = 0.88, *p* < 0.001). The present study indicates that when the renal tumor is small, values of split CT-GFR with and without renal tumor are very close (Supplementary Table 1). The difference between mean values of split CT-GFR with and without renal tumors in early renal parenchymal phase was 3.00 ± 2.30 ml.min^–1^.1.73 m^–2^ (*p* < 0.001). The difference between mean values of split CT-GFR with and without renal tumor in late renal parenchymal phase was 3.14 ± 3.13 ml.min^–1^.1.73 m^–2^ (*p* < 0.001). However, when the renal tumor is large, there is a big difference between the values of split CT-GFR with and without of renal tumor. For example, a 47-year-old man suffered from right sided renal tumor (Supplementary Figure 3). The volume of tumor is 114.17 cm^3^. The tumor-bearing kidney’s CT-GFR with and without renal tumor are 108.86 and 28.70 ml.min^–1^.1.73 m^–2^, respectively. Total CT-GFR without renal tumor (79.39 ml.min^–1^.1.73 m^–2^) is closer to the eGFR (105.19 ml.min^–1^.1.73 m^–2^) than the total CT-GFR with renal tumor (171.96 ml.min^–1^.1.73 m^–2^). Measurement split GFR without renal tumor by our approach can overcome the effect of tumor itself on GFR measurement.

Within this study, early and late renal parenchymal phase scans were performed to investigate the influence of time interval on the CT-GFR measurement. CT-GFR measured with early parenchymal phase showed a better correlation with eGFR compared to late parenchymal phase. The reason for this result may be that some contrast agents already excreted into the renal pelvis during the late parenchymal phase scan. It was not satisfied with the hypothesis of the two compartments model, and then affects the accuracy of CT-GFR measurements. Because a key assumption inherent to the Patlak analysis is that tracer entering the extravascular compartment should not leave that compartment during the sampling period (Hackstein et al., 2001). This assumption may be possible to explain that underestimation of CT-GFR value in late parenchymal phase. In addition, tubular function concentration and dilution of contrast medium could interfere the GFR calculation in late parenchymal phase (Hackstein et al., 2002). From our results, CT-GFR seems to underestimate eGFR. The reason for this may be explained from the physiological point of view. eGFR measures whole-body clearance of serum, which includes biliary excretion and should be somewhat higher than renal contrast media clearance, which measures the contrast media uptake in the kidney only in a measuring period.

As we know, radionuclide examination is considered the most widely used method for estimation of split GFR in clinical use (Russell and Dubovsky, 1989; Porpiglia et al., 2012), but this method fails to evaluate GFR without renal tumor preoperatively. Therefore, measurement of split and total GFR without renal tumor preoperatively needs further investigation. CT scan is routinely performed preoperatively to describe anatomy of vascular, kidney parenchymal, and tumor (Miles, 2003), especially for precise segmental artery clamping in laparoscopic partial nephrectomy (Xu et al., 2013). On CT enhancement scan, the injected iodinated contrast medium is not reabsorbed nor excreted at the renal tubules, which is the same as inulin clearance (Svaland et al., 1992). Therefore, some investigators described a technique for measuring GFR based on Patlak plot by enhancement CT (Blomley and Dawson, 1996; Miles et al., 1999). The Patlak method is a two compartments technique in which it is assumed that the contrast medium is fully trapped within the nephron space but the renal interstitial space is neglected (Hackstein et al., 2003). The problem of renal interstitial space happened in R-GFR examination as well. The Patlak plot analysis simplifies the transition of contrast medium through renal parenchymal and makes it possible for renal functional evaluation. However, this method has controversial concerning the CT scan protocol and imaging analysis procedures, and, therefore, are not adopted in clinical practice so far. Our study demonstrated a modified multiphasic CT scan protocol combined with homemade software to overcome this problem. Measurement of total CT-GFR in early parenchymal phase showed high agreement with eGFR. And there was no significant difference between relative split CT-GFR in early parenchymal phase and relative R-GFR. CT-GFR measured without renal tumor showed a better correlation with eGFR than CT-GFR measured with renal tumor. Our study demonstrated the feasibility of quantitative GFR by using multiphasic CT scan. There results suggested that a modified multiphasic CT scan protocol in combination with a homemade software appear to give the urologist another hint for assessment of split and total renal function preoperatively without the effect of the tumor. High levels of agreement of this software has been proved using intra- and inter-observer comparison, although the software is semi-automated and is partly user dependent. Furthermore, this has advantages for patients getting a routine scan, where additional examinations for kidney function estimation could be avoided.

However, our study has several limitations. Firstly, all patients in this study have no acute renal disorders. For patients with different degree of renal disorders, correlation and concordance between CT-GFR and GFR measurement of traditional method needs to be further explored. Secondly, the impact of the size and histology type of renal tumor on the measured values of CT-GFR needs further investigation. Thirdly, there will inevitably be a radiation burden to the patient higher than in conventional CT scan protocol. Some other improvements would also be meaningful, such as optimizing the model by using a lower dose scan. Moreover, we plan to research further on how to extend the method into magnetic resonance imaging GFR analysis. At last, for reference, 15 patients underwent radionuclide imaging, it still had not a relative big sample size with long follow-ups, which needs further study to prove the results.

## Conclusion

In conclusion, preoperative CT-GFR can be measured accurately with this modified multiphasic CT scan protocol combined with a homemade software based on two-point Patlak plot technique. This method is a non-invasive reliable modality providing not only morphological but also functional information for the patients with unilateral renal tumor. Further studies are required to confirm the clinical usefulness of the method in the residual function follow up for the patients with renal tumor partial nephrectomy.

## Ethics Statement

This study was carried out in accordance with the recommendations of name of the Nanjing Medical University’s Committee with written informed consent from all subjects. All subjects gave written informed consent in accordance with the Declaration of Helsinki. The protocol was approved by the name of the Nanjing Medical University’s Committee.

## Author Contributions

TW, YX, and LT: guarantor of integrity of the entire study and manuscript editing. LT, GY, TW, YX, WL, PS, and QL: study concepts and design. TW, YX, and WL: literature research. LT, GY, TW, YX, PS, and QL: clinical studies. LT, GY, TW, YX, and WL: experimental studies/data analysis. TW, YX, and WL: statistical analysis and manuscript preparation.

## Conflict of Interest Statement

The authors declare that the research was conducted in the absence of any commercial or financial relationships that could be construed as a potential conflict of interest.
