# Supplementary material for: Measurement of Glomerular Filtration Rate Using Multiphasic Computed Tomography in Patients With Unilateral Renal Tumors: A Feasibility Study
Source: Front Physiol. 2019 Sep 19;10:1209. doi: 10.3389/fphys.2019.01209 (PMC6761270; doi:10.3389/fphys.2019.01209)
Supplement: Supplementary file 1 [file Data_Sheet_1.docx]

**Supplemental files**

Supplementary figure 1 Image processing by software.


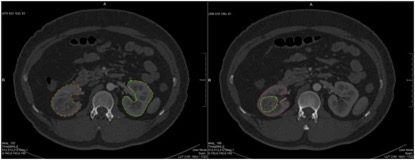


The left image shows the renal parenchymal area after surrounding structures including blood vessels and collecting system were excluded in axial image. The right shows space-occupying lesions were semi-automatically excluded from renal parenchymal area.

Supplementary figure 2 Bland-Altman test shows the results of CT-GFR measurement by the homemade software from the of 2 experienced radiologists.


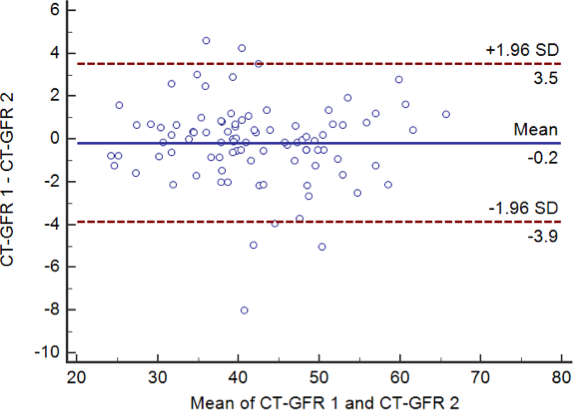


Supplementary figure 3 A 47-year-old man suffered from right sided renal tumor.


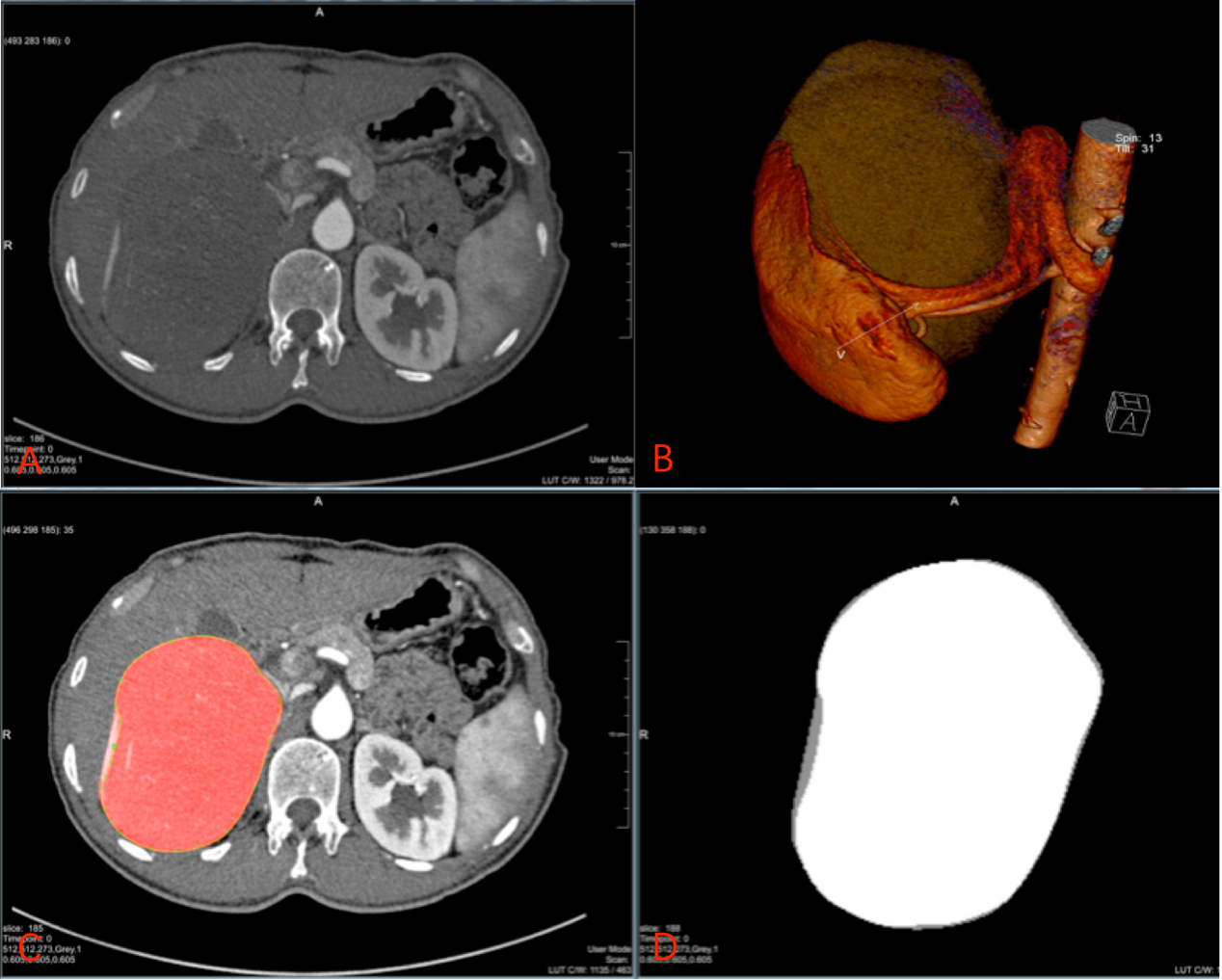


A showed the axial images of CT angiography. B showed volume rendering (VR) image of right kidney. C and D showed tumor was semi-automatically excluded from renal parenchymal area by homemade software.

Supplementary figure 4 CT images of region of interest in abdomen aorta at multiple time points


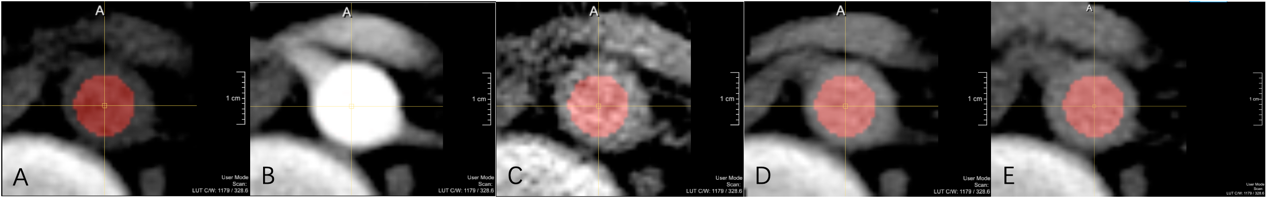


A showed the region of interest of the abdominal aorta in the phase of Bolus triggering.

B showed the region of interest of the abdominal aorta in the arterial phase.

C showed the region of interest of the abdominal aorta in the phase of Dynamic scans.

D showed the region of interest of the abdominal aorta in the Early parenchymal phase.

E showed the region of interest of the abdominal aorta in the Late parenchymal phase.

Supplementary table 1 Results of Statistical Comparison between Split CT-GFR of with Tumors and without Tumors

| Phase of renal parenchymal | GFR Measurement of tumor-bearing kidney | Paired *t* Test Difference | | | |
| --- | --- | --- | --- | --- | --- |
|  |  | ‾x ^*^(n=91)  (ml·min^-1^·1.73m^-2^) | | *t value* | *p value* |
| Early | CT-GFR(w) | 3.00±2.30 | 5.06 | | 0.000 |
|  | CT-GFR(wo) |  |  |  |  |
| Late | CT-GFR(w) | 3.14±3.13 | 3.89 | | 0.002 |
|  | CT-GFR(wo) |  |  |  |  |

CT-GFR(w)：Quantitative glomerular filtration rate with tumor based on the method of two point of Patlak plot；CT-GFR(wo)：Quantitative glomerular filtration rate without tumor based on the method of two point of Patlak plot

Supplementary table 2 Results of Statistical Comparison between Relative CT-GFR and Relative R-GFR of Split Kidney

| Relative GFR of split kidney | | Paired t Test Difference | | | Correlation Analysis | |
| --- | --- | --- | --- | --- | --- | --- |
|  |  | ‾x ^*^(n=15)  (ml·min^-1^·1.73m^-2^) | t *value* | *p value* | r *value* | *p value* |
| Non-tumor-bearing kidney | rR-GFR | … | … | … | … | … |
|  | Early rCT-GFR | -0.05±1.53 | -0.14^γ^ | 0.89^γ^ | 0.88^γ^ | <0.001^γ^ |
|  | Late rCT-GFR | 0.56±2.0 | 1.08^γγ^ | 0.30^γγ^ | 0.69^γγ^ | 0.004^γγ^ |
| Tumor-bearing kidney | rR-GFR | … | … | … | … | … |
|  | Early rCT-GFR | 0.05±1.53 | 0.14^λ^ | 0.89^λ^ | 0.88^λ^ | <0.001^λ^ |
|  | Late rCT-GFR | -0.56±2.0 | -1.08^λλ^ | 0.30^λλ^ | 0.69^λλ^ | 0.004^λλ^ |

^*^Data are‾x ± standard deviation

^γ^Data represent results of the comparison between rR-GFR and rCT-GFR in early renal parenchymal phase of non-tumor-bearing kidney.

^γγ^Data represent results of the comparison between rR-GFR and rCT-GFR in late renal parenchymal phase of non-tumor-bearing kidney.

^λ^Data represent results of the comparison between rR-GFR and rCT-GFR in early renal parenchymal phase of tumor-bearing kidney.

^λλ^Data represent results of the comparison between rR-GFR and rCT-GFR in late renal parenchymal phase of tumor-bearing kidney.

rCT-GFR：Quantitative relative glomerular filtration rate based on the method of two point of Patlak plot； rR-GFR：Quantitative relative glomerular filtration rate based on the method of radionuclide examination.

Supplementary table 3 Baseline clinical characteristics of patients.

| Characteristics | All patients (N=91) |
| --- | --- |
| Age (years) mean±SD | 58±12 |
| Male gender, n (%) | 58(64%) |
| Height(m) mean±SD | 1.66±0.07 |
| Weight(kg) mean±SD | 70±10.8 |
| Hypertension, n (%) | 33(36%) |
| Diabetes mellitus, n (%) | 10(11%) |
| Coronary artery disease, n (%) | 7(8%) |
| Arrhythmia, n (%) | 2(2%) |
| Hepatitis, n (%) | 3(3%) |
| Congenital heart disease, n (%) | 1(1%) |
| Chronic obstructive pulmonary disease, n (%) | 1(1%) |
| Tuberculosis | 2(2%) |
